# Modeling Permanent Deformations of Superelastic and Shape Memory Materials

**DOI:** 10.3390/jfb6020398

**Published:** 2015-06-11

**Authors:** Marco Fabrizio Urbano, Ferdinando Auricchio

**Affiliations:** 1 SAES Getters S.p.A., v.le Italia, 77, 20020 Lainate (MI), Italy; 2 Università di Pavia, Via Ferrata 9, 27100 Pavia, Italy; E-Mail: ferdinando.auricchio@unipv.it

**Keywords:** superelasticity, shape memory alloys, constitutive modeling, elastic modulus, functional fatigue

## Abstract

In this paper we propose a modification of the polycrystalline shape memory alloy constitutive model originally proposed by Souza. By introducing a transformation strain energy with two different hardening coefficients, we are able to take into account the effect of the martensitic transformation of unfavorably oriented grains occurring after the main plateau. By choosing a proper second hardening coefficient, it is possible to reproduce the correct stress strain behavior of the material after the plateau without the need of introducing a much smaller Young modulus for martensite. The proposed modification is introduced in the model comprising permanent deformation effects. Model results for uniaxial stress tests are compared to experimental results showing good agreement.

## 1. Introduction

Nitinol, a nearly equiatomic NiTi alloy, has been widely utilized in biomedical applications for more than 20 years. Peripheral stents, fixators, bone connectors are examples of Nitinol implants. Guide wires, endodontic files are examples of Nitinol medial or chirurgical tools.

Currently, more demanding applications of Nitinol are emerging: cardiac valves are an example.

Engineers, for the design of such devices, are more and more interested in constitutive models robust and easy to use but also able to accurately describe the material behavior. Typical loading paths contemplate a first string deformations, during which the material may overcome locally the plateau deformation and undergo permanent inelasticity, a deployment and a smaller deformation during the operating lifetime of the device. An accurate evaluation of stresses during the first deformations and the quantification of permanent inelasticity are needed for an accurate design of future Nitinol devices.

A 3D constitutive model first introduced by Souza *et al.* [1], further developed by Auricchio and Petrini [2], Auricchio *et al.* [3,4], and Barrera *et al.* [5], proved high stability and good predictive capabilities of both superelastic and shape memory behavior. However, the model provides a rather crude description of the material behavior at the end of the transformation where the real material shows a less steep stress-strain characteristic. This slope after the plateau has been traditionally described by introducing a different Young’s modulus for the martensite phase (see for example [6]). However, direct measurement of the elastic properties of Nitinol martensite and *ab initio* calculations (e.g., [7,8]) have shown that martensite have effective Young modulus similar and indeed higher than austenite. 

Recent investigations [9,10] have demonstrated that the softer behavior of polycrystalline NiTi after the transformation plateau has to be ascribed to the transformation of unfavorably oriented grains.

Taking advantage of these findings, here we propose a slight modification of the original Souza-Auricchio model consisting in the introduction of a double hardening coefficient in the transformation strain energy components of the pseudo potential. The first hardening coefficient is responsible for the main transformation plateau or temperature induced transformation strain. The second coefficient, which is much higher, describes the material behavior after the main transformation is completed.

After presenting the main model equations, we will compare the traditional model and the modified model in uniaxial stress conditions.

The proposed model variation will be subsequently introduced in the model with permanent deformation described in [5] and simulations of the model will be compared with experimental results.

## 2. 3D Phenomenological Model for Stress Temperature Induced Solid Phase Transformation

The model assumes the strain, **ϵ** and the absolute temperature, *T* as control variables and the second-order traceless transformation strain tensor ***e**^tr^* as internal variable. The quantity ***e**^tr^* has the role of describing the strain associated to the phase transformations.

Assuming a small strain regime, we express the free energy function for a polycrystalline SMA material through the following convex potential:

(1) $$\Psi \left(\epsilon ,e^{tr},T\right)=\Psi_{el}+\Psi_{ch}+\Psi_{tr}$$

Indicating with ***I*** the second-order identity tensor and introducing the standard decomposition:

$$\epsilon =\frac{\theta }{3}I+e$$

where θ and ***e*** are, respectively, the volumetric and the deviatoric components of the total strain, we describe the terms of Equation (1) as follows.

The elastic strain energy, due to thermo-elastic material deformation is:

(2) $$\Psi_{el}=\frac{1}{2}K\theta^{2}+G{\left\|e-e^{tr}\right\|}^{2}-3\alpha K\theta \left(T-T_{0}\right)$$

with *K* the bulk modulus, *G* the shear modulus, α the thermal expansion coefficient, and *T*_0_ the reference temperature. The operator ‖∗‖ indicates the Euclidean norm.

The chemical energy is:

(3) $$\Psi_{ch}=\beta \left(T\right)\langle T-T^{*}\rangle \left\|e^{tr}\right\|$$

with β(*T*) a function related to the dependence of the critical stress on temperature and 〈∗〉 the positive part of the argument.

Finally the transformation strain energy, due to the transformation-induced hardening, is set equal to:

(4) $$\Psi_{tr}=\{\begin{array}{c} \frac{1}{2}h_{1}{\left\|e^{tr}\right\|}^{2}\;if\;\left\|e^{tr}\right\|<\epsilon_{L}\\ \frac{1}{2}h_{1}\epsilon_{L}{\;}^{2}+\frac{1}{2}h_{2}{\left(\left\|e^{tr}\right\|-\epsilon_{L}\right)}^{2}\;if\;\left\|e^{tr}\right\|\geq \epsilon_{L} \end{array}$$

Starting from the pseudo-potential and following standard arguments, we obtain the model constitutive equations:

(5) $$\begin{array}{c} p=\frac{\partial \Psi }{\partial \theta }=K\left(\theta -3\alpha \left(T-T_{0}\right)\right)\\ s=\frac{\partial \Psi }{\partial e}=2G\left(e-e^{tr}\right)\\ X=-\frac{\partial \Psi }{\partial e^{tr}}=\{\begin{array}{c} s-\left(\beta \langle T-T^{*}\rangle +\;h_{1}\|e^{tr}\|\right)\frac{\partial \|e^{tr}\|}{\partial e^{tr}}\;if\;\|e^{tr}\|<\epsilon_{L}\\ s-\left(\beta \langle T-T^{*}\rangle +\;h\cdot \epsilon_{L}+h_{2}\left(\|e^{tr}\|-\epsilon_{L}\right)\right)\frac{\partial \|e^{tr}\|}{\partial e^{tr}}\;if\;\|e^{tr}\|\geq \epsilon_{L}\; \end{array} \end{array}$$

The model is then completed by introducing the associative evolution law:

(6) $${\dot{e}}_{tr}=\dot{\zeta }\frac{\partial F\left(X\right)}{\partial X}$$

and the Kuhn-Tucker conditions:

(7) $$\dot{\zeta }\geq 0,\;F\leq 0,\;\dot{\zeta }\cdot F=0$$

For the sake of simplicity, here we choose a symmetric yield function:

$$F={\left\|X\right\|}^{2}$$

In the present model no limit to the transformation strain is considered. This limitation could be easily overcome by introducing an indicator function as in the original model. It must considered, however, that reaching the full transformation would cause an increase of the slope in a monotonic stress strain test before plasticity takes place. This is not observed experimentally, suggesting that plasticity occurs before the complete martensite transformation occurs.

## 3. Comparison of Standard Model and the Proposed Model in Uniaxial Stress Tests

**Figure 1:**
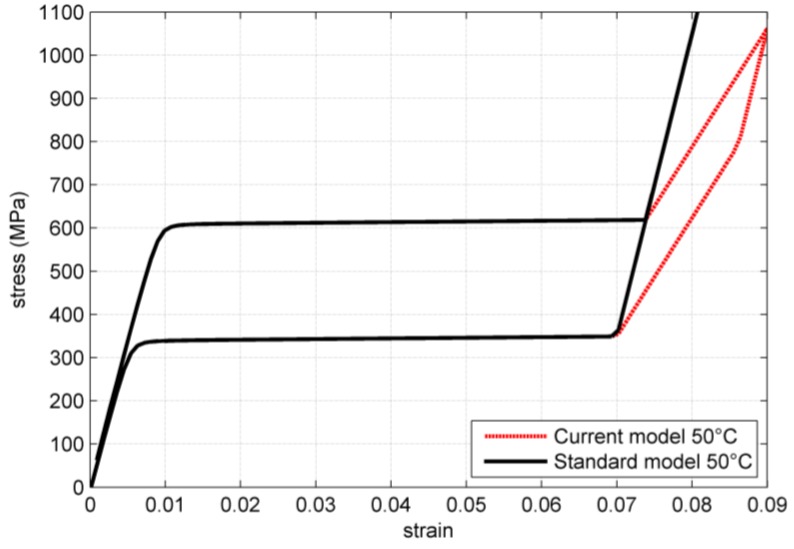
Comparison of standard model behavior in a uniaxial stress strain test starting from the parent phase.

The standard model and the modified model proposed are compared by simulating a uniaxial stress strain test. The results are shown in Figure 1. The parameters utilized for the simulations are summarized in Table 1.

**Table 1:** Parameters utilized for the model comparison.

Parameter	Value
*G*	2.63 × 10^4^ MPa
*K*	6.86 × 10^4^ MPa
α	1 × 10^−5^
β	4.4 MPa/K
*h*_1_	100 MPa
*h*_2_	3 × 10^4^ MPa
*ϵ_L_*	$0.065\cdot \sqrt{3/2}$
*R*	110 MPa
T*	235 K

Starting from the rest condition in the parent phase the two models behave identically up to the end of the plateau. After reaching the maximum transformations strain ϵ_*L*_, the standard model behaves elastically with a young modulus identical to the one of the parent phase. On the contrary, in the modified model the transformation continues, but with a much higher hardening. This section of the stress-strain characteristic can be described by the following equation:

(8) $$\sigma_{11}=\sqrt{\frac{3}{2}}\;2G\left(\alpha_{3}\|e\|+\alpha_{2}\right)$$

where

$$\alpha_{1}=\beta \langle T-T_{0}\rangle +\left(h-h_{2}\right)\epsilon_{L}$$

$$\alpha_{2}=\;\frac{\alpha_{1}+R}{2G+h_{2}}$$

$$\alpha_{3}=\frac{h_{2}}{2G+h_{2}}$$

During unloading, the modified model firstly behaves elastically. Like the original model, the proposed modified model assumes identical elastic modulus of the austenite and the martensite phase. When the critical stress is reached, the reverse transformation starts, initially with the higher hardening, assuming that the reverse transformation of the unfavorably oriented grains is the first to take place. When the transformation modulus is lower than ϵ_*L*_, the reverse transformation proceeds with the lower hardening coefficient. At this point the two models again provide an identical solution.

The proposed model variation, consisting in the modified term of the pseudo-potential Ψ_*tr*_ described in Equation (4), can be easily introduced in the model contemplating permanent inelasticity described in [5].

The model equations will not be re-proposed here. We will only recall the new quantities introduced in the model to account for permanent inelasticity, and the additional parameters.

Permanent inelasticity is described by two new internal variables, the plasticity tensor ***e**_pl_*, the functional fatigue tensor ***q***, modeling the irreversible processes induced by microscopic and macroscopic stresses. Increasing ***q*** prevents the material from fully recovering generic external deformations. The thermodynamic force associated to the functional fatigue tensor is identified by the tensor ***Q***. For the model definition it is necessary to define the limit function ξ, that now must comprehend both the thermodynamic force ***X*** and ***Q***, the functional fatigue hardening *H* and the coefficient *a*, describing the ratio between the plasticity tensor and the functional fatigue tensor.

For the simulations with permanent inelasticity we have adopted the limit function:

(9) $$\xi =max\left\{{\left\|X,Q\right\|}_{k,n}-R,0\right\}$$

where ${\left\|\nu_{1},\nu_{2}\right\|}_{k,n}={\left({\left|\nu_{1}\right|}^{n}+k{\left|\nu_{2}\right|}^{n}\right)}^{\frac{1}{n}}$. The following quantities (Table 2) complete the parameter set reported in Table 1.

**Table 2:** Parameters set utilized for the model comprising permanent inelasticity.

Parameter	Value
*H*	100 MPa
*A*	1
*N*	5
*k*	0.07

**Figure 2:**
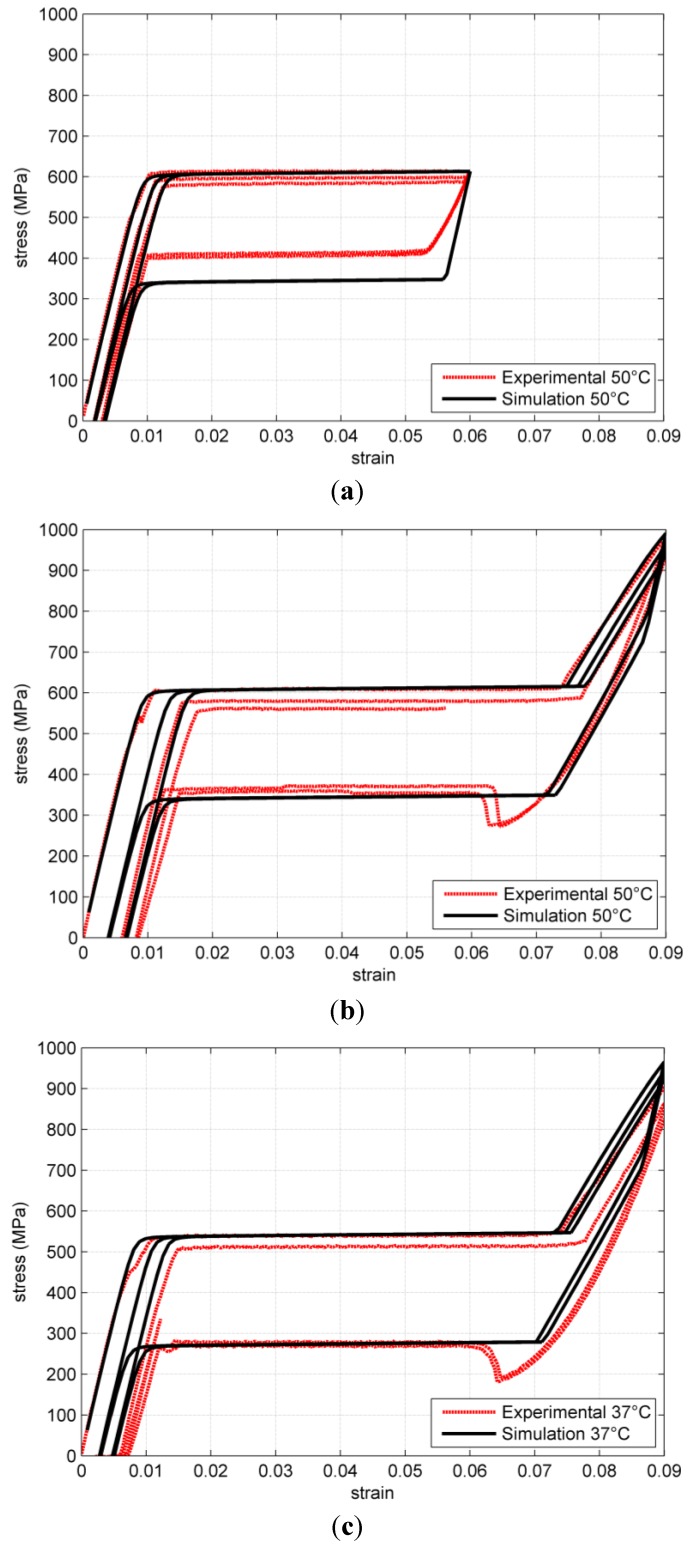
Comparison of simulated and experimental results for stress strain uniaxial tests of superelastic Nitinol at different temperatures. (**a**) stress-strain at 50 °C up to 6% strain; (**b**) stress train at 50 °C up to 9% strain; (**c**) stress-strain at 37 °C up to 9% strain.

**Figure 3:**
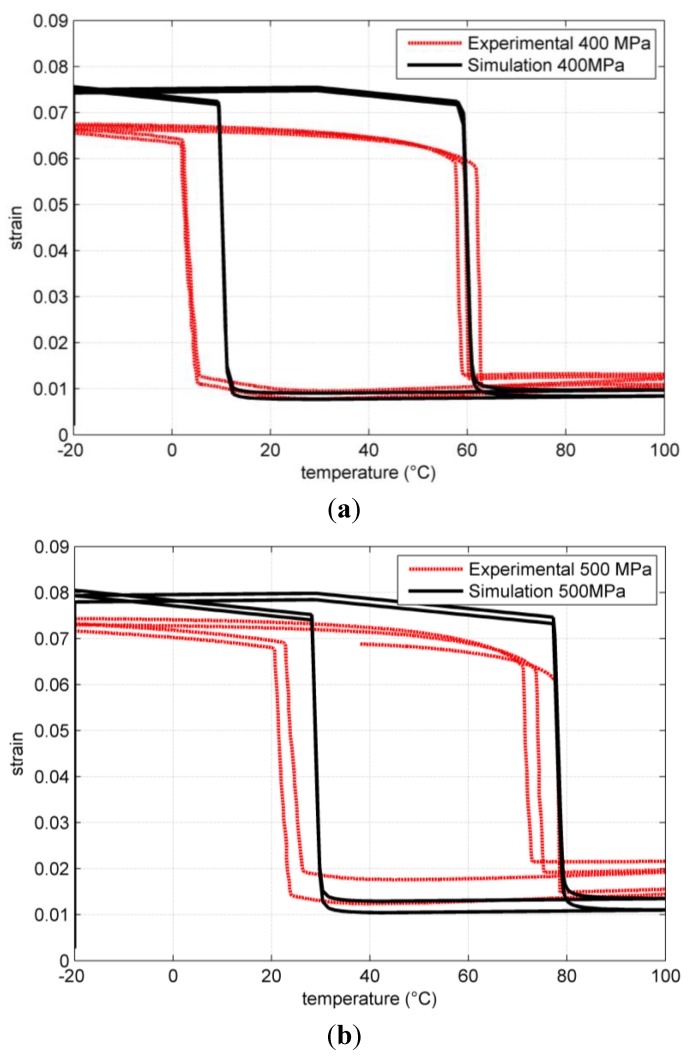
Comparison of simulated and experimental results for temperature loops under uniaxial tensile load at different tensile stresses. (**a**) 400 MPa; (**b**) 500 MPa.

The adopted parameters are not obtained with a rigorous fitting procedure but provide a reasonable matching with experimental results. A comparison is shown in Figure 2 and Figure 3.

Experiments have been carried on a standard 0.3 mm diameter superelastic wire provided by Memry. Stress strain test and temperature loops at constant load have been performed on SMAq, an internally constructed characterization tools [11].

**Figure 4:**
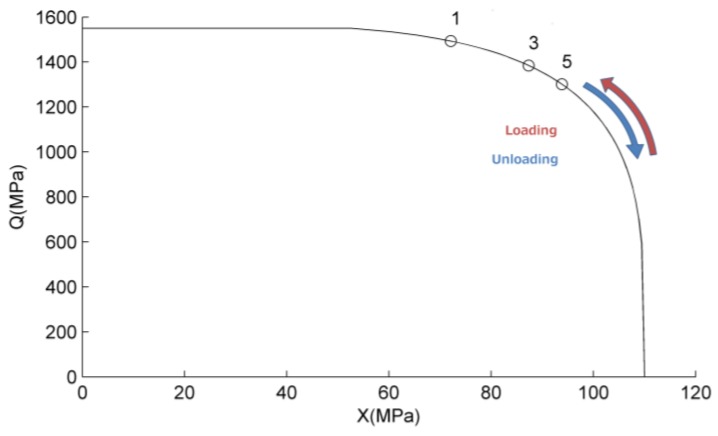
Thermodynamic force position at the end of the tension load step at 50 °C. Points 1, 3 and 5 refer to the first, second and third cycle respectively.

Several loading conditions are provided. In order to understand the internal variable interplay, we start analyzing a stress strain test at 50 °C up to 9% deformation (Figure 2b), with the help of Figure 4, where the thermodynamic force status is represented. 

Upon loading, when the plateau stress is reached the thermodynamic forces are located on the limit boundary (black line of Figure 4) and the internal variables evolve according the flow rule. The limit curve is traveled counterclockwise by the force coordinates until unloading. Points 1, 3 and 5 represent the last point reached by the material after the first, second and third loading cycle. On subsequent cycles the *Q* component of the force is lower, due to accumulated hardening, for the same total deformation conditions.

On the limit curve, the tensor $\dot{q}$ and ${\dot{e}}_{tr}$ are parallel and the constant of proportionality equals the tangent of the normal to the ξ(X,Q) function. Thus, when the material in on point 3 of Figure 4, the ratio between the increment in ***q*** and the increment in ***e_tr_*** is lower than in position 4. This explains why the ***q*** tensor increment, that can be appreciated looking at the residual strain at zero stress, decreases at increasing cycles.

In Figure 2b the results for stress strain loop inverted at lower strain. The residual strain is lower than in the previous case because the thermodynamic forces stay in the lower part of the limit curve, where the normal is practically parallel to the *X* axis.

At lower temperature (37 °C), the model correctly predicts a decrease in the plateau stress. 

With the same parameter set, we have performed temperature loops at constant load simulations and these are compared to experimental results in Figure 3. The model transformation strain is in this case slightly overestimated. However, the model succeed in correctly predicting the transformation temperature shift with load and the residual strain.

## 4. Conclusions

We have described a modification of the Souza-Auricchio model accounting for the residual phase transformation occurring after the main plateau of polycrystalline Nitinol. This phase transformation involves unfavorably oriented grains and it is rendered by introducing a second hardening coefficient in the transformation energy component of the pseudo-potential.

This modification can be easily implemented also in the model version contemplating permanent inelasticity. The modified model has been tested by comparing simulated and experimental uniaxial tests, with satisfactory agreement.

Further investigations are needed in order to investigate the model behavior in more complex loading conditions. 
